# Non-antibiotic selection systems for soybean somatic embryos: the lysine analog aminoethyl-cysteine as a selection agent

**DOI:** 10.1186/1472-6750-9-94

**Published:** 2009-11-18

**Authors:** Suryadevara S Rao, Lewamy Mamadou, Matt McConnell, Raghuveer Polisetty, Prachuab Kwanyuen, David Hildebrand

**Affiliations:** 1 Plant & Soil Sciences, University of Kentucky, Lexington, KY 40546, USA; 2 USDA-ARS/Crop Sciences, NC State University Raleigh, NC 27607, USA

## Abstract

**Background:**

In soybean somatic embryo transformation, the standard selection agent currently used is hygromycin. It may be preferable to avoid use of antibiotic resistance genes in foods. The objective of these experiments was to develop a selection system for producing transgenic soybean somatic embryos without the use of antibiotics such as hygromycin.

**Results:**

When tested against different alternate selection agents our studies show that 0.16 μg/mL glufosinate, 40 mg/L isopropylamine-glyphosate, 0.5 mg/mL (S-(2 aminoethyl)-L-cysteine) (AEC) and the acetolactate synthase (ALS) inhibitors Exceed^® ^and Synchrony^® ^both at 150 μg/mL inhibited soybean somatic embryo growth. Even at the concentration of 2 mg/mL, lysine+threonine (LT) were poor selection agents. The use of AEC may be preferable since it is a natural compound. Unlike the plant enzyme, dihydrodipicolinate synthase (DHPS) from *E. coli *is not feed-back inhibited by physiological concentrations of lysine. The *dapA *gene which codes for *E. coli *DHPS was expressed in soybean somatic embryos under the control of the CaMV 35S promoter. Following introduction of the construct into embryogenic tissue of soybean, transgenic events were recovered by incubating the tissue in liquid medium containing AEC at a concentration of 5 mM. Only transgenic soybeans were able to grow at this concentration of AEC; no escapes were observed.

**Conclusion:**

Genetically engineered soybeans expressing a lysine insensitive DHPS gene can be selected with the non-antibiotic selection agent AEC. We also report here the inhibitory effects of glufosinate, (isopropylamine-glyphosate) (Roundup^®^), AEC and the ALS inhibitors Exceed^® ^and Synchrony^® ^against different tissues of soybean

## Background

A prerequisite for most current plant genetic engineering procedures is the ability to produce transgenic plants. The process of producing transgenic plants often requires effective means for identifying and selecting transgenic cells and tissues. An important method of soybean regeneration is somatic embryogenesis. Through somatic embryogenesis, genetic engineering of soybean has proved to be a powerful technique for improving seed compositions including the oil for enhanced edible and industrial purposes [1-5]. Somatic embryogenesis (SE) is the process whereby embryos develop from either microspores or somatic tissues. In contrast to the cotyledonary node or other adventitious (non-embryogenic) regeneration systems, the somatic embryo based system appears to be mostly derived from single cells in the epidermal layers of the primary somatic embryos which increases the chances of obtaining non-chimeric regenerated plants[6,7]. Another potential advantage of the somatic embryos (SE) system is that they are good targets in many cases of seed specific traits since they can be analyzed at the mature soybean somatic embryo stage prior to the zygotic embryonic stage, thus saving labor and time. Embryogenic tissues can be proliferated by subculture on solid proliferation (MSD20) medium or liquid suspension culture medium[8,9]. One of the well-established soybean transformation procedures is the particle delivery system (gene gun) bombardment of somatic embryos [10-12]. Christou et al. [13] were the pioneers in the area of biolistic transformation of soybeans using immature seed meristems. The bombarded SEs are selected with molecules that can be inactivated by genes encoded on the introduced DNA. A selection agent that has been used successfully is the antibiotic hygromycin and this has become the standard for selection of soybean SE. However, the presence of antibiotic resistance genes in food may not be desirable due to the potential incorporation of such genes by human pathogens. There would not be selective pressure for horizontal transfer [14] of genes such as the bacterial DHPS reported here to human pathogens. Herbicide resistance genes may be of less health concern although they can be transferred to wild plant relatives including weeds potentially reducing the efficacy of weed control by the herbicides. Few studies have been reported on using herbicide selectable agents/markers like glyphosate, glufosinate, and ALS herbicides for selecting soybean somatic embryos. However, herbicides such as glyphosate and glufosinate have been extensively used in the transformation and selection of several crops such as maize, tobacco, wheat, rice, alfalfa, etc. and soybeans with the cotyledonary node adventitious regeneration system [15]. Aragão, Rech and co-workers [16,17] report on use of a modified acetohydroxy acid synthase gene to bombard embryonic axes and select soybeans transformed by a cotyledonary node system with the ALS inhibiting imidazolinone herbicide, Imazapyr.

Glyphosate is a herbicide that has been evaluated in this study as a potential selection agent. Glyphosate has been proven to be a potent broad-spectrum herbicide that interferes with the biosynthesis of aromatic amino acids by specifically inhibiting the enzyme 5-enolpyruvylshikimate-3-phosphate (EPSP) synthase (the sixth enzyme) of the shikimate pathway.

Herbicidal compounds that inhibit acetolactate synthase (ALS) which is also called acetohydroxy acid synthase (AHAS) the first enzyme involved in the biosynthesis of the branch-chain amino acids thereby resulting in the death of the selected plant tissues [18-20] have also been evaluated in this study.

Phosphinothricin (PPT) ammonium or L-glufosinate ammonium, the active component of the herbicide Liberty^® ^also called "basta", is a structural analogue of glutamate. As a consequence, PPT acts as a glutamate analogue and inhibits glutamine synthetase leading to plant cell death [21-23]. The bar gene has been used as the selectable marker coupled with glufosinate as a selection agent with the *Agrobacterium tumefaciens*-cotyledonary node transformation system for soybeans [15] succeeded in germ-line transformation events at frequencies up to 3% using a selection regime of 5 mg/L glufosinate during the shoot initiation stage and 2 mg/L during shoot elongation with soybean cotyledonary nodes transformed with *Agrobacterium*. Simmonds and Donaldson [24] report on the selection of two transgenic soybean lines by selecting somatic embryo cultures expressing a phosphinothricin N-acetyltransferase gene with glufosinate.

**Figure 1:**
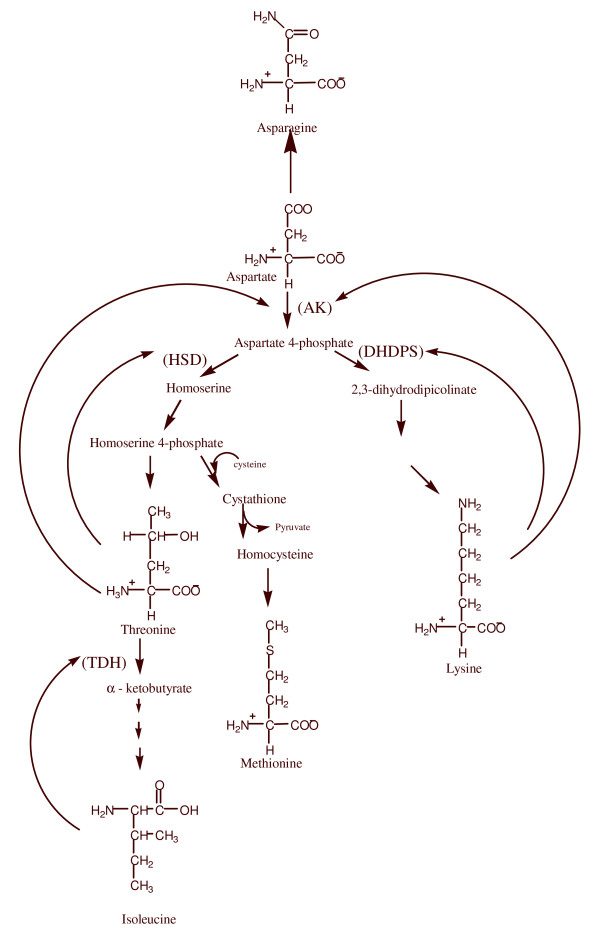
**A diagram of the aspartate-family biosynthesis pathway**. All the key enzymes and their products are indicated. Curved arrows represent feedback inhibition by the end product amino acids. AK: aspartate kinase; DHPS: dihydrodipicolinate synthase; HSD: homoserine dehydrogenase; TDH: threonine dehydrogenase.

Some alternate selectable markers can also be chosen from the pool of information on other amino acid biosynthetic pathways that are available. One such example is the regulatory enzyme aspartate kinase (AK) of the aspartate family pathway that leads to the synthesis of lysine, threonine, methionine and isoleucine and that can be a point of feedback regulation (Figure 1). This pathway is regulated by several feedback inhibition loops [25]. AK consists of several isozymes that are feedback inhibited by lysine and threonine [25,26]. Treatment with millimolar concentrations of lysine plus threonine (LT) strongly inhibit the growth of a wide range of plant species [25,27-29] due to inhibition of AK activity causing starvation for methionine [25,27-29] Mutants selected for resistance to LT possess AK genes encoding desensitized enzymes.

Dihydrodipicolinate synthase (DHPS) is another important regulatory enzyme of this pathway that is extremely sensitive to feedback inhibition by lysine and is the major limiting factor for the synthesis of this amino acid. The relatively poor efficiency of lysine synthesis makes plants generally highly sensitive to the toxic lysine analog, S-2 aminoethyl L-cysteine (AEC), which competes with lysine for incorporation into proteins [28,30,31]. Only lysine overproducing plants, or plants with defective uptake of this analog, are resistant to AEC. The use of desensitized *E. coli *AK and *E. coli *DHPS insensitive to lysine (I_50 _being 1 mM lysine) as a plant selection agent has been reported for potatoes [25,32].

The objective of these experiments was to develop a selection system for producing transgenic soybean somatic embryos without the use of antibiotics such as hygromycin. Given the concerns regarding glyphosate tolerance being developed by weeds Behrens et al. [33] alternate selection systems which can be developed into potential herbicides is a future necessity Our results indicate that glufosinate, (isopropylamine-glyphosate) (Roundup^®^), AEC and the ALS inhibitors Exceed^® ^and Synchrony^® ^could be useful as selection agents for soybean somatic embryos. However, lysine and threonine were found to be poor selection agents. We were successful in developing a novel non-antibiotic system to select transgenic soybean somatic embryos using the *E. coli dapA *gene. We also report here the inhibitory effects of glufosinate, (isopropylamine-glyphosate) (Roundup^®^), AEC and the ALS inhibitors Exceed^® ^and Synchrony^® ^against different tissues of soybean.

## Methods

### Plant material

Soybeans [*Glycine max *(L.) Merrill cv. 'Jack'] were grown in the greenhouse at University of Kentucky, Lexington under a 16 hr photoperiod. Pods with immature seeds were surface sterilized by immersing for 30 sec in 70% 2-iso-propyl alcohol followed by a 10 min immersion in 25% of 10% commercial bleach (1.5% hypochlorite) with a few drops of Liquinox (detergent) per L. The pods were then rinsed three times in sterile water for 5 min each time. Immature seeds 3-6 mm in length were removed from the pods. The hilum-side containing the embryonic axis was cut off and discarded. Then the two cotyledons were pushed out from the seed coat, separated and placed with the abaxial side (round side) down on MSD40 medium [12,34]. Cultures were then incubated at 25°C at a 23 hr photoperiod and low light intensity, 5-10 *μ*Em^-2^s^-1^. Globular staged somatic embryo (SS embryos) clusters were harvested from the explant tissues 4 to 6 weeks after induction and then placed on MSD20 solid medium [12] for a period of one month for proliferation. Embryogenic tissues were then transferred from MSD20 to FNL (Finer and Nagasawa "lite") "liquid medium" [9] for further proliferation. Suspension cultures were agitated at 100 rpm and maintained with a 2 week subculture period at 25°C with a 23 hr photoperiod. For each of these experiments conducted, 200 mg of globular stage soybean somatic embryos were used as starting materials in 125 mL Erlenmeyer flasks containing 30 mL of FNL (Finer Lite) culture medium.

### Selection media

**Figure 2:**
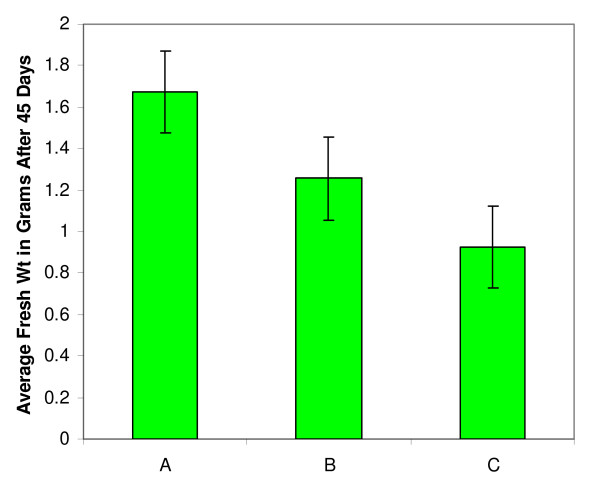
**Growth increase in full-strength vs. reduced-strength media**. Average fresh weight in grams of soybean somatic embryos after cultured for 45 days in different media. A: control in Finer Lite (FNL) medium (Finer et al., 1999). B: 1/5 FNL medium and C: 1/5 FNL medium without asparagine. N = 3

The growth of the somatic embryos was tested at different media concentration levels to determine the concentration that reduced the growth of the somatic embryos most. Normal FNL medium was used either at normal full concentration (where indicated) or at 1/5 concentration with or without asparagine. 1/5 FNL medium with asparagine will be referred to as 1/5 normal FNL medium (Figure 2). About 200 mg of globular somatic embryo cultures (in triplicate) were used as starting materials in 125 mL Erlenmeyer flasks containing 30 mL of culture media (full concentration, 1/5 concentration with asparagine and 1/5 concentration without asparagine). No difference in growth was observed in full-strength medium with and without asparagine so for further studies were conducted in full-strength medium without asparagine. For each type of medium, a sample in triplicate (as control) was set and transferred twice (every 15 days) into fresh medium. At the end of this period, the somatic embryos were drained and excess moisture removed and weighed to determine the rate of growth in each culture medium. The average weight of soybean somatic embryos from each culture medium was then recorded after 45 days.

#### Determination of AEC and LT concentrations for selection on solid medium

Along with the liquid medium (FNL) selection we also tried the solid (D20) medium selection for soybean somatic embryos for LT and AEC. It has been a common observation among the soybean somatic embryo transformation scientists that while solid medium selection is relatively consistent liquid medium based selection is more rapid (personal communication with John Finer). To select the soybean somatic embryos (SSE) on solid medium, non-transformed globular stage SSE were plated on D20 medium [8] with different concentrations of AEC and LT. The concentration at which the SS embryos turned brown and stopped any further proliferation was determined.

### Vector construction

The coding sequence of *E. coli dapA *([35]; Genbank accession number M12844) was amplified from DH5α cells using the primers 5'GGC GCC ATG TTC ACG GGA AGT3'and 5'TCT AGA TTA CAG CAA ACC GGC ATG3' for the sense and antisense strands. A Kas I restriction site was added to the forward primer while the reverse primer was designed with an Xba I site. A pea (*Pisum sativum *L.) rbcs (Rubisco small subunit) chloroplast transit peptide (TP) [36] was amplified from pBluescript vector containing TP using the forward 5'AAG CTT ATG GCT TCT ATG ATA TCC3' primer and a reverse 5'GGC GCC GCA CTT TAC TCT TCC ACC3' primer with Hind III and Kas I restriction sites. A Hind III/Kas I digested pea rbcS transit peptide was ligated to Xba I/Kas I digested *dapA *DNA. The ligated product was amplified using the transit peptide forward and *dapA *reverse primers and cloned into the PGEM T-vector. The product was sequenced using T7 and SP6 sequencing primers to confirm the ligated product. A 1.1 kbp Hind III/Xba I digested fragment was cloned into a pKYLX71 vector [37] digested with the same enzymes to insert the transit peptide fused *dapA *under the CaMV 35S promoter. The whole cassette was digested with EcoR I/Pst I and ligated to pCambia 1201 (Genbank accession number AF234293) digested with the same restriction enzymes. Prior to this ligation the hpt gene (conferring hygromycin resistance) from pCambia 1201 was removed by digesting with Xho I and self ligating the plasmid.

### Microprojectile bombardment

Slightly mashed green embryo clumps were placed in the center of a moist filter paper in sterile petri plates (approximately 100 to 150 mg of somatic embryos per plate) and partly dried in a laminar flow hood for 15 min prior to bombardment. Transformation was carried out via particle bombardment with a gene gun (DuPont PDS1000; Bio-Rad Laboratories, Hercules, and CA) by gold/DNA micro projectile preparations as described by [12]., for nine shots, 25 μg of DNA was used to coat 7.5 mg of 0.6 μm gold spheres. Cultures were bombarded at 10,687 kPa (1,550 psi) helium gas pressure under a 91 kPa (27 in of Hg) vacuum, at a shooting distance of 11 cm from the rupture disk to target tissue. Immediately after bombardment, embryogenic cultures were placed on D20 proliferation medium without any selection agent for seven days.

### Selection and regeneration of transformants

Bombarded globular soybean somatic embryos (SSE) that were cultured on D20 were transferred (approximately 100 clumps of 0.3-0.4 cm diameter per plate) onto selection medium containing 5 mM AEC. After 3-4 weeks, visibly growing clumps were moved to fresh selection medium. After approximately 12 weeks, AEC resistant SSE clumps (masses) that appear green in color were transferred to FN lite [8] liquid proliferation medium. For maturation, the AEC resistant embryogenic clusters were transferred from FN Lite into 500-mL Erlenmeyer flasks (5 clusters per flask) containing 100 mL of liquid MS0M3 [9] medium. Prior to transfer into a flask, each embryogenic cluster was gently pressed with a spatula to partially separate the individual globular-stage embryos. At four weeks, embryos were checked with glucuronidase (GUS) stain (Jefferson et al., 1987) Some of the embryos were desiccated as described by [9]. Ten to 12 matured embryos were placed in a 100 × 15 mm Petri dish and sealed with Nescofilm. To allow gradual desiccation of embryos over a period of 5-7 days, a small piece (approximately 1 cm^3^) of MS0 medium was placed in the middle of the plate away from the embryos. Desiccated embryos were germinated on 1/5^th ^MS medium.

### GUS staining

Leaves and flowers of transgenic plants were placed overnight in vials containing the GUS assay buffer [50 mM Na-phosphate, 500 mM potassium ferrocyanide, 10 mM EDTA, 1 mM X-gluc (5-bromo-4chloro-indolyl-glucuronide) and 0.05% Triton X-100, pH 7.0] and incubated overnight at 37°C [38]. The assay buffer was decanted and the tissues were bleached in 95% EtOH and photographed.

### DNA isolation

Genomic DNA was isolated from matured soybean somatic embryos [39] by homogenizing 8-10 embryos in 1 ml of extraction buffer (100 mM Tris-HCl, pH 8.0, 20 mM EDTA, 0.5 M NaCl, 0.5% [wt/vol] sodium dodecyl sulfate [SDS], and 0.5% [vol/vol] β-mercaptoethanol). The homogenate was extracted with 500 μl of a phenol/chloroform/isoamyl alcohol mixture (25:24:1) and centrifuged at 12,000 × *g *for 5 min. The aqueous phase was collected, mixed with 1 μl of 10 mg/ml of RNase A, and incubated at room temperature for 20 min. The samples were re-extracted with an equal volume of phenol/chloroform/isoamyl alcohol (25:24:1), followed by two re-extractions with chloroform/isoamyl alcohol (24:1). DNA was precipitated by centrifugation after adding 2.5 volumes of ethanol. Precipitated DNA was washed with 70% ethanol, dried, and resuspended in 100 μl of sterile water.

### PCR

100 ng of genomic DNA was used to amplify the *dapA *and lysC genes from transgenic tissues of soybean in a polymerase chain reaction. The primers used for *dapA *amplification were as follow: 5'GGC GCC ATG TTC ACG GGA AGT3' for the forward primer and 5'TCT AGA TTA CAG CAA ACC GGC ATG3' for the reverse primer. The PCR conditions were 94°C for 2 minutes; 30 cycles at 94°C, 30 seconds; 55°C, 30 seconds; 68°C, 1:30 minutes and a final extension at 68°C for 8 minutes.

### RNA isolation

RNA was isolated using the Trizol Reagent (Life Technologies, GibcoBrl). Soybean somatic embryos of each individual line that were grown for 3 weeks in maturation medium were homogenized in 1 mL Trizol reagent, transferred into microcentifuge tubes, and incubated for 5 minutes at room temperature. 0.2 mL chloroform per 1 mL of Trizol was added; tubes were vigorously shaken for 15 seconds and incubated at room temperature for 3 minutes. Samples were centrifuged at 12,000 × g for 15 minutes at 2 to 8°C. The upper aqueous phase containing RNA was collected in fresh tubes; RNA was precipitated with 0.5 mL of isopropyl alcohol and samples incubated at room temperature for 10 minutes. Samples were centrifuged at 12,000 × g for 10 minutes, and the RNA pellet was washed once with 1 mL of 75% ethanol. Samples were vortexed and centrifuged at 7,500 × g for 5 minutes. The RNA pellet was air-dried, then dissolved in RNase-free water and incubated for 10 minutes at 60°C. The concentration of RNA was estimated by reading the absorbance at 260 nm. The 260/280 ratio of the RNA was 1.9 to 2.0. The quality of RNA was checked using 1.2% agarose gels. Samples were stored at -80°C until used.

### RT-PCR

Total RNA extracted from the leaves of the AEC resistant soybean somatic embryos was used to amplify *dapA *cDNA. Reverse transcription was performed for the synthesis of the first-strand cDNA using oligo dT as prescribed by manufacturers of the Kit (Sigma-Aldrich Corporation, St. Louis, MO, USA) in a 20 μL reaction at 48°C for 45 minutes. A 2 μL aliquot of the RT reaction was used for PCR with the following profile: 94°C for 2 minutes; 30 cycles at 94°C, 30 seconds; 55°C, 30 seconds; 68°C, 1:30 minutes and a final extension at 68°C for 8 minutes. The primers used were the same as used in PCR reaction described for transgene amplification.

### Selective agents

#### The following selective compounds were used at various concentrations

-S-(2-aminoethyl)-L-cysteine hydrochloride or AEC (Sigma chemical company, St Louis, MO (USA), L-lysine (Biosciences, inc. La Jolla, CA (USA)), L- threonine (Sigma), Liberty herbicide (AgrEvo USA), Exceed (Syngenta Crop Protection, Inc. Greensboro, NC USA), Synchrony (Dupont Wilmington, DE USA), Hygromycin from Invitrogen, Carlsbad, USA), N-(Phosphonomethyl)-Glycine (Sigma). Roundup (Monsanto, St. Louis, USA)

Exceed or 3-[4,5-bis (difluoromethoxy)-pyrimidin-2-yl]-1-(2-methoxycarbonyl-phenylsulfonyl) urea is a combination of two different compounds (Primisulfuron methyl 2-[[4,6-bis (difluoromethoxy)-2-pyrimidinyl]amino]sulfonyl]benzoate and Prosulfuron 1-(4-methoxy-6-methyl-triazin-2-yl)-3-[2-(3,3,3-trifluoropropyl)-phenylsulfonyl]-urea) that are blended together; and Synchrony is also a combination of two different chemicals or compounds (Thifensulfuron methyl 3-[[[(4-methoxy-6-methyl-1,3-5-triazin-2-yl) amino]carbonyl]amino]sulfonyl]-2-thiophenecarboxylate and Chlorimuron ethyl 2-[[[(4-chloro-6-methoxy-2-pyrimidinyl) amino] carbonyl] amino] sulfonyl] benzoate) that are blended together [19].

Roundup^® ^which contains N-phosphonomethyl-glycine predominantly in the form of the isopropyl amine salt at 1.92% is a herbicide known to affect EPSPS (5-enolpyruvylshikimate-3-phosphate synthase) pathway was tested for selection.

### Lysine analyses

#### Sample preparation

For total lysine analysis, 100 mg of ground samples were homogenized in 2.0 mL Milli-Q (Millipore, Bedford, MA) water in a 16 × 100 mm glass tube with Brinkman (Westbury, NJ) Polytron homogenizer equipped with a 7-mm generator for 1 min at a speed setting of 5. Five μL of homogenates were transferred to 6 × 50 mm glass tubes that had been acid-washed with 6 N HCl for 24 hr. Samples were then lyophilized. Gas-phase hydrolysis was performed according to [40] with modifications. The hydrolyses were done in duplicate. Lyophilized samples were placed in each evacuated glass container with 200 μL of 6 N HCl and 10% phenol in the bottom. Evacuation in 25-30 in-Hg pressure alternated with nitrogen flushing was performed at least three times. Samples were hydrolyzed at 110°C *in vacuo *for 24 hrs. After hydrolysis, samples were dried under a vacuum and stored in a freezer until derivatization and HPLC analysis. For free lysine analysis, 100 mg of ground samples were extracted in 1.0 mL of 50 mM phosphate buffer, pH 8.0, for 1 hr. Samples were centrifuged at 15,000 × g for 10 min. Aliquots of 5 μL of the supernatants were lyophilized and used for derivatization and HPLC analysis in the same manner as for total lysine analyses. This also was done in duplicate.

#### Lysine analyses

All samples including amino acid standards were neutralized by adding 20 μL of a 2:2:1 mixture of ethanol:water: Triethylamine (TEA) (v/v), and mixing well with a vortex. They were dried under vacuum. Derivatization was performed by adding 20 μL of mixture of 7:1:1:1 ethanol:water:TEA: Phenylisothiocyanate (PITC) (v/v), and mixing well by vortexing. The reaction between PITC and the hydrolysate to produce phenylthiocarbamyl (PTC) amino acids was allowed to complete for 20 min at room temperature. Samples were then completely dried under vacuum. PTC amino acids in each sample and standards were dissolved by vortex mixing with 500 μL of 5 mM Na_2_HPO_4 _buffer, pH 7.4 containing 5% acetonitrile. It was then filtered through 0.2 μm membrane. Reconstitution of samples was done one at a time due to the PTC amino acid sensitivity to light and ambient temperature. Ten μL of samples were injected and analyzed with a Hewlett-Packard (Agilent Technologies, Wilmington, DE) series 1050 HPLC system equipped with column heater, autosampler, variable wavelength detector series 1100, and ChemStation data acquisition software controller. The reverse-phase column used was a Pico-Tag (Waters Corp., Milford, MA) with an in-line column filter. The column temperature was maintained at 38°C. The PTC amino acids were separated and eluted by a gradient resulting from mixing buffers A and B. Buffer A consisted of 150 mM CH_3_COONa.3H_2_O, 0.05% TEA, and 6% acetonitrile, pH 6.4. Buffer B consisted of 6:4 acetonitrile:Milli-Q water (v/v). Both buffers were sparged with helium for 10 min before use. The flow rate was 1 mL/min throughout, and the gradient consisted of the following profiles: 100% A at start, 80% A and 20% B at 5.5 min, 54% A and 46% B at 10 min, 100% B at 10.5 to 12.5 min, 100% A at 13 min. The PTC amino acids eluted from column was detected at 254 nm and recorded. The column was regenerated and equilibrated with buffer A for 10 min. A new and freshly reconstituted sample was injected and analyzed every 23 min.

## Results

### ALS Herbicide selection

**Figure 3:**
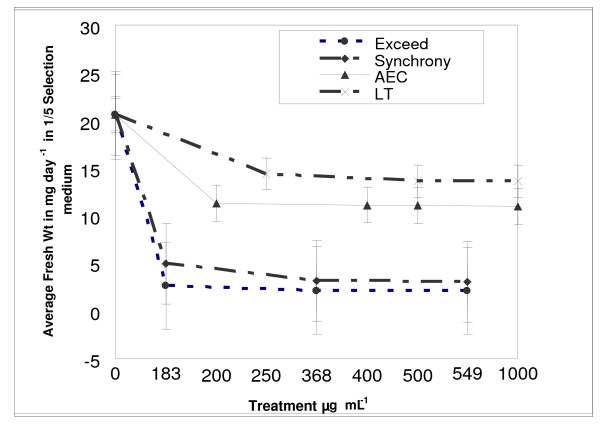
**Average fresh weight of soybean somatic embryos (SSE) tissues cultured in different 1/5 (FNL) culture media without asparagine containing: Lysine plus Threonine, two ALS inhibitor herbicides (Exceed and Synchrony) and a lysine analog (AEC)**. The medium containing Lysine plus threonine (**x**) was found to be less effective than the other compounds (with about 70% growth inhibition obtained after 120 days). The Exceed containing medium (◆) was found to be more efficacious than the one containing Synchrony (●). However, in either case it took four weeks for all SSE tissues growth to be completely inhibited. With the medium containing the lysine analog AEC (▲), 100% of the SSE tissues ceased growth after a period of four months at all treatment levels. n = 3

Two different types of ALS inhibitors, Exceed and Synchrony were tested. Both herbicides were found to be heat labile and work better in the lower nutrient medium rather than in the normal FNL medium (Figure 3).

### Glyphosate/Roundup^® ^selection

Roundup^® ^was found to be effective at the concentration level of around 730 μM (123 mg/L) in the full medium with or without asparagine. It works even better at a concentration as low as 245 μM (41.4 mg/L) in the 1/5 medium minus asparagine and at the concentration level of 400 μM (or 68 mg/L) in the 1/5 normal FNL medium. The active ingredient of Roundup (N- (phosphonomethyl)-glycine) from Sigma (St. Louis, MO), needed to be used at a concentration of at least 980 μM (165.6 mg/L) and a longer time was required to inhibit the growth of the tissue.

### Liberty^® ^or basta selection

**Figure 4:**
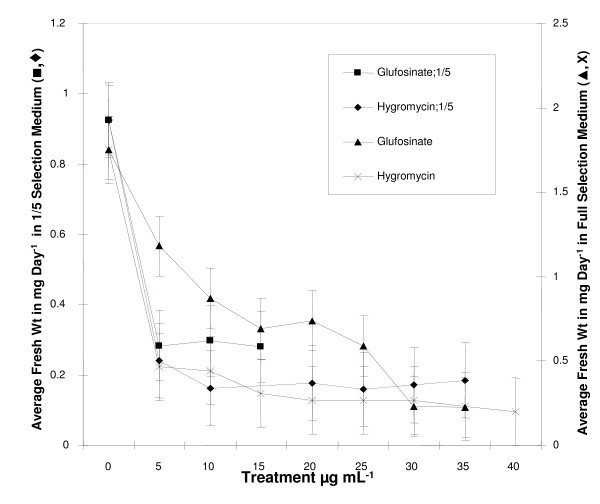
**Average fresh weight of soybean somatic embryos (SSE) tissues after culture for 2 to 5 weeks in different culture media**. Growth of more than 95% of the SSE was inhibited within three weeks of their selection in the 1/5 FNL minus asparagine selection medium containing the selection agent glufosinate or Basta (■). In the medium with hygromycin (◆), growth inhibition of the SSE was observed within the first week of selection. However, in the full selection medium total growth inhibition was obtained within four weeks at the treatment above 20 μg/mL for both selection agents: hygromycin (**x**) and glufosinate (▲). n = 3

This selection was first carried out in normal FNL medium. The following concentrations (5, 10, 15, 20, 30, and 35 μg/mL) or (25.3, 50.5; 75.8; 101; 151.5 and 176.7 μM) were used with and without asparagine (Figure 4). One week after incubation, between 45 - 50% of the initial somatic embryo tissues had lost their green color and developed a brownish color. The growth of more than 95% of the somatic embryo tissues was inhibited three weeks later and the growth of the remaining 5% was also inhibited after an additional two weeks. The selection in 1/5 concentration FNL medium without asparagine was then carried out with the following concentration: 0.16, 0.33, and 0.5 μg/mL or (0.81; 1.67; and 2.5 μM) (Figure 4). After two transfers lasting an average of 15 days (between each), the growth of all the somatic embryo tissues was found inhibited. The only difference was the growth inhibition of the embryos started earlier (during the first 7 days after selection), while in the normal FNL medium, the inhibition started to be noticeable only during the second week.

### Hygromycin selection

Selection with hygromycin was first conducted in full medium concentrations of 5 to 40 μg/mL or (10 to 76 μM). The treatments with 20 μg/mL (40 μM) and above were found to be effective after two transfers of 15 days each (100% growth inhibition) (Figure 4). The selection was also done in 1/5 normal FNL medium and 1/5 FNL medium without asparagine. In the selection medium without asparagine, the growth of 95% of the materials was inhibited during the first week of selection at all the treatment levels. In comparison with the 1/5 full selection medium somatic embryo tissue growth was inhibited at treatment levels 20 μg/mL (38 μM) and higher (Figure 4). Selection in 1/5 medium without asparagine was complete in less than two weeks while with the full medium it took more than three weeks.

### Lysine + threonine (LT) Effects

The selection of soybean somatic embryos with lysine and threonine was initiated in normal FNL medium without asparagine at concentrations of 2.5, 5 and 10 mM (0.75, 1.6 and 3.2 mg/mL). This experiment lasted for a period of more than five months with transfers to fresh medium every 15 days. At the end of the second transfer (i.e. after a month), signs of growth inhibition were observed with 10 - 20% of the embryos.

After a period of 120 days, about 70% of the embryos growth was inhibited and the remaining were still green (Figure 3). The preliminary experiments with LT in regular FNL medium never resulted in the growth inhibition of 100% tissues (30-40% of the tissues remained green even after 4 months). The failure of the LT to inhibit the growth of the SSE led us to check whether these compounds will be more effective in reduced strength FNL medium. However, the same concentration of 2,4-D as in the normal FNL medium was kept constant to help maintain embryo proliferation. The 1/5 FNL medium without asparagine was selected to carry out subsequent experiments (Figure 3). Thus, the LT experiment when done in this new environment with respect to the same conditions (surfactant and without asparagine) appeared to be more effective than when it was conducted in the normal FNL medium. However, even in this new environment, we observed that after a 4 months period, only about 70-80% of the embryos stopped growing and the other 20-30% remained green.

### AEC (Lysine analog) treatment

At first, the selection was conducted with the lysine analog, AEC, on FNL medium without asparagine but with the addition of surfactant (methylated soybean oil). All the materials stopped growing after seventeen weeks (~4 months) and with a total of 8 transfers. The requirement of a high amount (5 mM) and long duration led to the analysis to determine whether this chemical is more effective in reduced strength 1/5 normal FNL medium. In comparison to LT, AEC appeared to be a better candidate as an alternative selection agent. With the 1/5 medium, all of the somatic embryos stopped growing after 13 weeks at the 5, 7.5 and 10 mM or (1, 1.5, and 2 mg/mL) treatments (Figure 3) and growth was more than 95% inhibited at the 2.5 mM treatment which was not effective in the full concentration medium.

### LT and AEC selection of different soybean tissues

**Figure 5:**
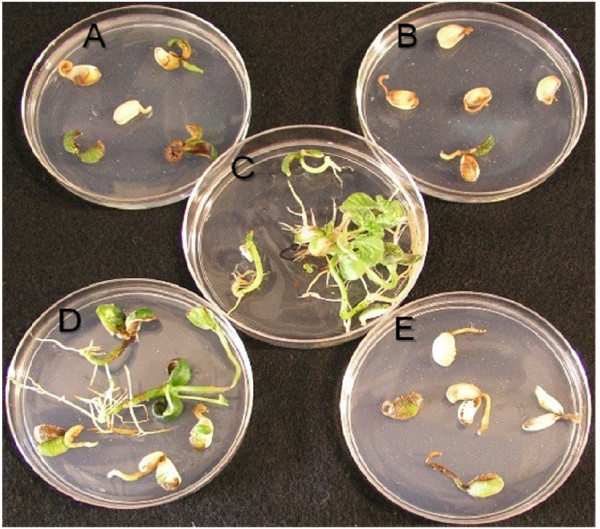
**Inhibition of soybean seed germination at different AEC concentrations; A. 2.5 mM, B. 5 mM, C. 0 mM, D. 500 μM, E. 1.5 mM**.

**Figure 6:**
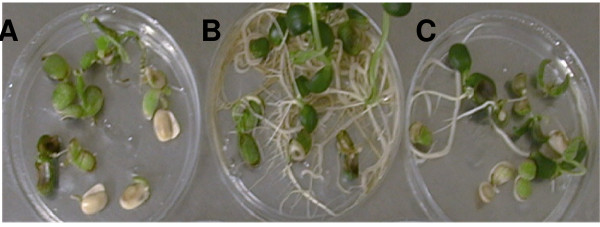
**Inhibition of soybean seed germination at different lysine + threonine (LT) concentrations; A. 10 mM, B. 0 mM, C. 5 mM**.

**Figure 7:**
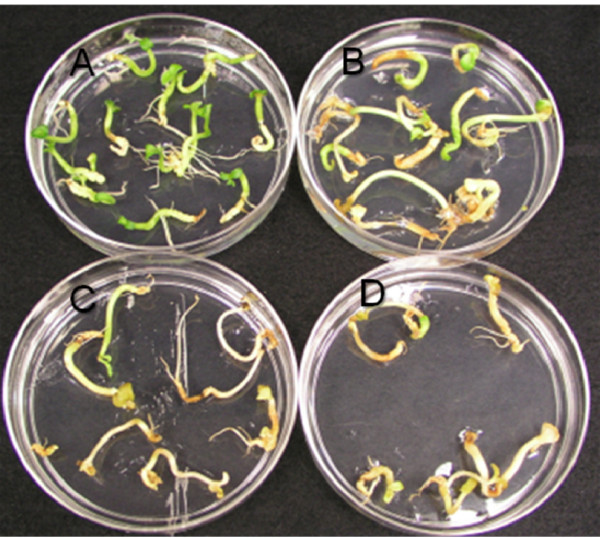
**Inhibition of soybean mature somatic embryo germination at different AEC (S-(2aminoethyl)-L-cysteine); A. 0 μM, B. 100 μM, C. 250 μM, D. 500 μM AEC**.

**Figure 8:**
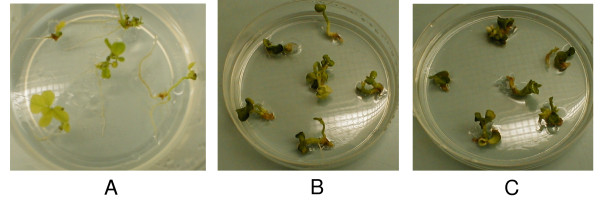
**Inhibition of soybean mature somatic embryo germination at different LT (Lysine + Threonine) concentrations, Control (A), 5 mM (B) and 10 mM (C)**.

**Figure 9:**
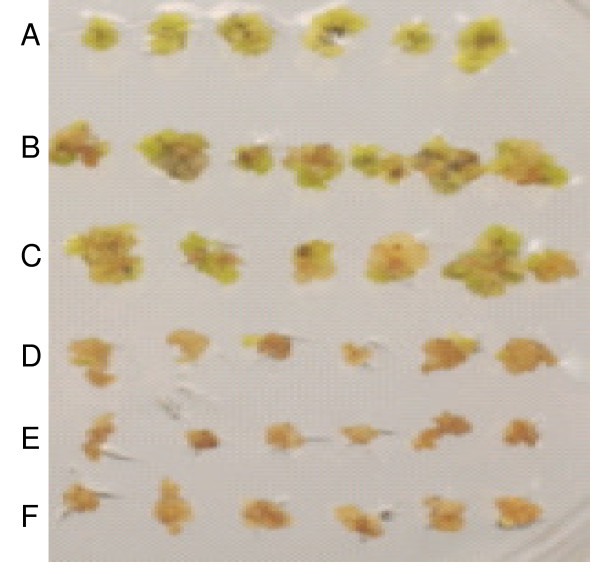
**Proliferation inhibition of non transformed soybean globular embryos at different AEC (S-(2aminoethyl)-L-cysteine) concentrations**. Concentrations by row from top to bottom of AEC are (in mM) 0, 0.5, 1, 1.5, 2.5 and 5 are represented by A, B, C, D, E and F respectively.

**Figure 10:**
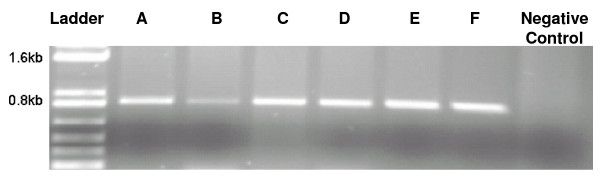
**RT-PCR of different soybean somatic embryo transformants expressing *E. coli *DHPS**. A, B, C, D, E, and F are different transgenic lines.

**Figure 11:**
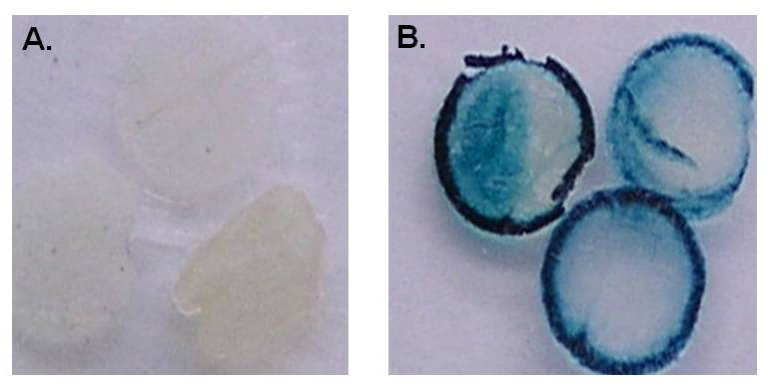
**GUS staining of control (A) and transgenic soybean leave discs (B)**.

We examined the relationship between the dose of LT or AEC administered and growth inhibition to test whether soybean can be selected with LT and AEC on plates. Seed germination results showed that these compounds can inhibit germination at certain concentrations. Germination of soybean seeds on MS plates containing 0-5 mM AEC, showed that 1.5 mM AEC completely inhibited germination (Figure 5). Similarly, 10 mM LT completely inhibited soybean seed germination (Figure 6). Interestingly, only 250 μM AEC was required to inhibit germination of matured soybean somatic embryos while 5 mM LT was required for the same purpose (Figure 7 &8). 5 mM AEC totally prevented the proliferation of globular stage somatic embryos compared to control, which are the target for biolistic transformation (Figure 9) on D20 plates. Since LT, even at 10 mM did not inhibit somatic embryo proliferation, soybean transformation with lysine insensitive AK was not performed, instead only the *dapA *construct was used for soybean somatic embryo transformation. The lysine insensitive AK was used by Perl et al. [25] as a selection marker to select potato. The potato shoot and root regeneration was successfully inhibited by LT in the selection medium. The over expression of a lysine insensitive AK prevented the cell death normally caused by the deprivation of methionine in such cases. Since soybean somatic embryos are never totally inhibited by LT the initial idea of using lysine insensitive AK as a SSE selection marker was shelved. The *dapA *transformed AEC resistant putative transgenic somatic embryos on D20 medium are shown in (data not shown). However, we never obtained any positive transformants in liquid medium selection. These embryos were further proliferated, matured, and analyzed by GUS staining. All of the lines analyzed were GUS positive. These lines have also been found to have the introduced dapA gene. To test whether the introduced gene was being transcribed total RNA from soybean somatic embryos was subjected to RT-PCR with *dapA *Figure 10). Soybean *dapA *transgenic lines were obtained from three different independent experiment using both AEC and hygromycin selection systems. Approximately 100 masses (200-250 mg) of 0.3-0.4 cm diameter per plate were used for one shot and a total of 9 shots per experiment. 5-7 transgenic events were obtained per experiment with the AEC system and these results are also similar to results obtained with solid medium hygromycin selection system[41]. The transgenic T1 and vector control plants were taken to the greenhouse and grown under the same condition as the parent Jack. Different tissues of transgenic plants were analyzed for GUS activity (Figure 11). The PCR results of *dapA *transgenics showed a 3:1 segregation of the introduced dapA gene in the T2 progeny. No difference in the growth pattern and seed development was seen between transgenic and wild type plants.

Dap A transgenic, AEC resistant soybean plants had normal phenotypes (data not shown). No abnormalities in the flower development and seed production were noticed in the transgenic plants. When analyzed for their free and total lysine contents, no significant difference was found in the seed of T2 progeny when compared to the control and the parent Jack (Table 1).

**Table 1:** Free and total lysine levels of Dap A transgenic, vector control (VC) and parental soybean seeds (Jack).

	Free Lysine (pmoles/mg)		Total Lysine (g/100 g protein)	
Sample	mean	SE	mean	SE
VC2 7	32.7	6.0	6.6	0.1

VC2 8	36.0	0.3	5.5	0.2

VC2 9	30.3	2.6	5.6	0.04

VC2 10	33.9	1.0	5.9	0.3

H2 10	36.3	0.3	6.0	0.2

B2 19	24.3	0.7	6.8	0.1

E2 5 1	37.6	2.2	6.5	0.1

Jack	28.1	2.1	6.0	0.4

Means and standard errors of two or more replications

## Discussion

Cellular selection is often necessary in tissue culture and molecular biology work. Most systems use a specific dominant selectable marker to enable the recovery of transgenic tissues once a selection agent is proven to be efficient. Hygromycin is the standard selection agent for soybean somatic embryo tissues. However, possibilities exist in using alternative selection agents and markers in place of hygromycin. A potential group of herbicides, amino acids, and their analogs have been tested as possible selection agents to further improve transgenic soybean somatic embryo selection. Most herbicides act by affecting biochemical processes including photosynthesis, essential amino acid biosynthesis, and other biochemical processes that are unique to plants or distinctly different from other organisms. Resistance to herbicides or feed-back inhibition by amino acids which results from one or more mutations of the genes involved in these pathways can provide alternative means of selection.

The selection medium salts were diluted and tested at different concentration levels of nutrients with and without asparagine: from full strength to 1/2, 1/3, 1/4, 1/5 and 1/10 concentrations (data was not shown). The concentration of 2,4-D was kept constant in all these different selection media. At the 1/10 medium concentration, the soybean SE stopped growing completely and growth inhibition occurred within the first week presumably from starvation. The minimum selection medium concentration that sustains an acceptable growth of the SSE (increasing by almost 50% relative to full-strength media) was found to be at 1/5 of its normal concentration (Figure 2).

Most of the selection agents tested in this study were found to be more effective in the 1/5 culture medium compared to the full-strength medium. Of the different compounds tested, in either normal FNL or 1/5 normal FNL medium, Liberty^® ^(glufosinate) was found to be effective at the lowest concentration level 2.5 μg/mL. Using the cotyledonary node method of soybean transformation Zhang et al. [42] observed 3-5 μg/ml glufosinate was sufficient to prevent shoot initiation. These amounts are similar to the concentrations of glufosinate required to inhibit SSE proliferation.

In the case of glyphosate, our experiments showed that the salt form of the chemical works better than the acid form. Glyphosate, in the salt form, was found to work even better in 1/5 selection medium with and without asparagine and at a concentration as low as 400 and 240 μM when compared to the full concentration medium (730 μM). This could be due to the relative insolubility of the compound in water as well as the lack of surfactant (s) found with the Roundup Ultra formulation (data not shown).

Four classes of ALS-inhibiting herbicides including sulfonylureas, imidazolinones, triazolopyrimidines, and pyrimidinyl thiobenzoates are found to inhibit the first enzyme involved in the biosynthesis of valine, leucine, and isoleucine. In our experiments, two types of sulfonylurea herbicides were used: Exceed and Synchrony. Although formulated for two different crops, Exceed^® ^for corn and Synchrony^® ^for soybean [19], we found that both ALS-inhibiting herbicides tested inhibited soybean somatic embryo growth after a period of one month. However, Exceed^® ^was found to be more effective than Synchrony^®^. Furthermore, the sequence of phytotoxic mechanisms with these chemicals remains unclear [19]. The growth inhibition of somatic cells by these compounds (Exceed^® ^and Synchrony^®^) could be a result of any of the following factors: direct depletion of the end products, depletion of intermediates of the pathway for some critical processes, or from buildup of a toxic intermediate [43]. In our experiments we found that the two ALS -inhibiting herbicides (Exceed and Synchrony) were found to be less effective in normal FNL medium than in the reduced concentration medium.

The lysine analog AEC and LT have been used as agents for crop improvement since the 1980's [28]. These chemicals were used to select tissues and plants that overproduce either threonine or lysine. Compared to the other lysine analogues AEC was found to be more effective in inhibiting plant growth [31,44]. The effect of lysine on the two key enzymes, AK and DHDPS, can be mimicked by AEC in higher plants [31,44-46].

In our study, AEC proved to be effective in 1/5 normal FNL medium and in a shorter period of time. However, it was found to be as effective when used in 1/5 D20 or full strength D20 solid media (Figure 9). In this experiment, lysine-threonine was observed to be less effective with the soybean somatic embryos.

### Differential absorption of LT and AEC

Our results showed that different concentrations of LT and AEC inhibit different tissues from soybean to differing degrees (Figures 5, 6, 7, 8, 9 and 11). These results are more pronounced with AEC. Our selection results also showed that concentrations of AEC needed for inhibiting soybean tissue growth varied greatly. 2.5 mM AEC was required for the inhibition of soybean seed germination while 5 mM was best for inhibiting somatic embryo proliferation (Figures 5 &9). Interestingly, only 250 μM was needed for the inhibition of soybean somatic embryo germination (Figure 7). This shows that different soybean tissues responded to different amounts of AEC. Our results with soybean were in conformity with some of the AEC concentrations reported in mutagenic studies. Depending on the plant species, prior studies indicate that the AEC concentration for growth inhibition varied: 1.5 mM and 1 mM for rice calli and rice anther-derived cell lines and 1 mM for sweet potato shoot inhibition[47]; 0.25 mM for seeds of *Arabidopsis thaliana *[48]; and 125 μM for potato shoot formation [25]. Growth inhibition of all of these tissues at different concentrations is an indication of possible existence of a differential metabolism mechanism of AEC depending on their metabolic state. Similar results were reported by [49] for the mutagenic agent sodium azide. They concluded that sodium azide was differentially metabolized by maize callus and germinating maize embryos using different detoxification and repair mechanisms. The higher sensitivity shown by some tissues like germinating soybean somatic embryos may be the result of more than one factor. It is known that AEC inhibits AK and DHPS [25]. AEC was also found to inhibit cell growth irreversibly by competing with lysine for incorporation into protein, leading to an altered protein processing and folding, and may also account for its cytotoxic effects [50,51].

### AEC selection of transgenic SS embryos

All soybean somatic embryos selected with AEC were GUS and PCR positive. Our selection results suggest that AEC and the *dapA *gene can make a good selection system for proliferating soybean somatic embryos. Efficiency of transformation with the *dapA *gene and selection with AEC in soybeans is similar to that of hygromycin (data not shown), where 3-5 clumps is positively transformed for every 9 shots. Due to the use of solid selection medium, the growth of the embryos with the AEC selection system was a little slower compared to hygromycin liquid selection system. This is consistent with Samoylov et al. [9]. On solid D20 selection medium 8-10 weeks led to the identification of AEC resistant green transgenic clumps, whereas 6-8 weeks were required for hygromycin selection. However, it is important to point out that AEC selection of transgenic soybean somatic embryos in liquid medium is not satisfactory compared to solid medium although the kill curve studies clearly showed lesser amounts of AEC are needed for growth inhibition in liquid medium (Figure 3). The opposite is seen with hygromycin selection in which selection on solid medium is much slower (~5 months) than with liquid medium (10-12 weeks) (at least from our observations). To our knowledge, the only plant species previously selected using this *dapA *selection system was potato [25]. Perl et al. [25] have shown that shoot and root generation of control potato tubers were completely inhibited at 0.1 and 0.15 mM concentrations of AEC. The *dapA *transgenic plants showed resistance at these concentrations. However, at higher concentrations of AEC (0.3 mM) transgenic plants were also susceptible. No AEC selection is reported for any other plants expressing *dapA*.

For biolistic experiments on an average for a single shot 25 globular masses were used. When slightly mashed and matured each clump can give rise to ~50 individual matured embryos. The total number of individual matured embryos obtained from 25 clumps will be ~1000. Each experiment generally consists of 9 such shots by gene gun totaling ~9000 embryos. With AEC selection on the solid medium ~5 transgenic clumps for 9000 embryos were recovered ≈0.05% transformation efficiency. As mentioned earlier a similar number of transgenic plants were obtained with hygromycin as the selection agent. Zhang et al. [42] using basta for selection for *Agrobacterium*-mediated soybean cotyledonary node transformation observed a 0 to 3% transformation efficiency based on GUS expression. With the same method using hygromycin as a selection marker Olhoft et al. [52] reported a 0.7% efficiency. They improved the transformation efficiency to 16.4% by using a super virulent strain of *Agrobacterium *in presence of thiol compounds in the medium. Compared to the *Agrobacterium*-based cotyledon node method the biolistic transformation of somatic embryos is low. However, advantages including early testing of seed-specific traits and proliferation of large number of transformed embryos that can ensure the establishment of plants with more surety can balance the drawbacks. Arago et al. [53] reported a transformation efficiency of 0.7% when *Phaseolus vulgaris *embryonic axes were bombarded.

When chimeric *dapA *and *lysC *genes were introduced into tobacco under a CaMV 35S promoter an increase in free lysine content was reported in leaf tissues. The authors speculated the use of tissue specific promoters to increase lysine content in the seeds [54,55]. In the later studies Karchi et al., 1994 showed the role of lysine catabolism in the prevention of lysine accumulation tobacco seeds when *dapA *was expressed under a seed-specific promoter. Falco et al. [56] increased the soybean and canola seed free lysine and total lysine content by expressing *Corynebacterium dapA *gene under a seed-specific promoter. The unaltered seed lysine levels in the present study might be the result of the promoter used to express the dapA gene. Since the primary objective of this study was to develop *dapA *as a selection marker for soybean somatic embryos a 35S promoter was used.

### LT selection

The inhibition of plant growth by LT is reported to be due to poor methionine biosynthesis [25]. It was reported in the literature that 2-3 mM LT is inhibitory to seed germination [57]. Our soybean seed germination results with LT confirm this observation. However, for inhibition of soybean seeds, a little higher concentration of LT is needed. However, the growth of soybean somatic embryos was never totally inhibited even at 10 mM LT.

One of the primary concerns of genetically modified (GM) crops is that antibiotic resistant genes could be transferred to pathogenic microbes in the gastrointestinal tract or soil rendering them resistant to treatment with such antibiotics [58]. With biolistics, the DHPS can be shot with the genes of interest, thus eliminating the need for antibiotics in the transgenic soybeans. In conclusion genetically engineered soybeans expressing a lysine insensitive DHPS gene can be selected with the non-antibiotic selection agent AEC. Using this strategy one can exclude antibiotic selection by introducing the expression cassette of lysine resistant DHPS through biolistics with a cassette expressing a gene of desired trait into plants.

## List of abbreviations

AEC: S-(2 aminoethyl)-L-cysteine; AHAS: acetohydroxy acid synthase; AK: aspartate kinase; ALS: acetolactate synthase; DHPS: dihydrodipicolinate synthase; EPSPS: 5-enolpyruvylshikimate-3-phosphate synthase; FNL: Finer & Nagasawa "lite" liquid medium for proliferation; HSD: homoserine dehydrogenase, LT: lysine plus threonine; MSD20: Murashige & Shoog "proliferation medium all with 20 mg/L 2,4-D; PEP: phosphoenolpyruvate; PPT: phosphinothricin ammonium or L-glufosinate ammonium; SE: somatic embryo; SSE: soybean somatic embryo; TDH: threonine dehydrogenase).

## Authors' contributions

SR conceived the idea of AEC selection of soybean somatic embryos, made the vector constructs, did the biolistics, selection of the transgenic tissue, and established T1 generation plants. LM did the kill curves, GUS analysis of transgenic material, seed chip analysis of the T3 generation to find out the *dapA *segregation ratio. RP did the RT-PCR and PCR of the transgenic plants to establish transgenic nature of the material. MM did the tobacco transformation that was used as control and helped with the photographs. Free and total lysine analysis was done by PK. The work was done in DH's lab at the University of Kentucky under his overall guidance and supervision. All authors read and approved the final manuscript.
